# Long Range Raman-Amplified Distributed Acoustic Sensor Based on Spontaneous Brillouin Scattering for Large Strain Sensing

**DOI:** 10.3390/s22052047

**Published:** 2022-03-06

**Authors:** Shahab Bakhtiari Gorajoobi, Ali Masoudi, Gilberto Brambilla

**Affiliations:** Optoelectronics Research Centre, University of Southampton, Southampton SO17 1BJ, UK; sbg1v17@soton.ac.uk (S.B.G.); gb2@orc.soton.ac.uk (G.B.)

**Keywords:** distributed acoustic sensor, DAS, DVS, phase-OTDR, Brillouin-OTDR, strain sensor

## Abstract

A Brillouin distributed acoustic sensor (DAS) based on optical time-domain refractometry exhibiting a maximum detectible strain of 8.7 mε and a low signal fading is developed. Strain waves with frequencies of up to 120 Hz are measured with an accuracy of 12 με at a sampling rate of 1.2 kHz and a spatial resolution of 4 m over a sensing range of 8.5 km. The sensing range is further extended by using a modified inline Raman amplifier configuration. Using 80 ns Raman pump pulses, the signal-to-noise ratio is improved by 3.5 dB, while the accuracy of the measurement is enhanced by a factor of 2.5 to 62 με at the far-end of a 20 km fiber.

## 1. Introduction

High sensitivity and long sensing range of Distributed Acoustic Sensors (DASs) have made them ideal devices for sensing vibrations of elongated structures such as pipelines, railways, and subsea cables [1,2,3,4,5,6]. The sensitivity of DAS systems based on Phase-sensitive Optical Time-domain Reflectometry (ϕ-OTDR) has seen a continuous improvement over the past decade, reaching levels as low as several tens of p$\varepsilon /\sqrt{Hz}$ [7,8,9,10,11,12,13,14]. Although the lower strain limit of ϕ-OTDR based DAS (ϕ-DAS) systems are extended to sub nε levels, the ceiling of the strain range of these systems is limited to several tens of με due to the trade-off between the maximum sampling rate and the length of the sensing fiber [15,16] (detailed explanation of this trade-off is provided in Section 2). This limitation restricts the use of ϕ-DAS in applications that require measurement of high strain-rates over long distances such as analysis of large-magnitude earthquakes or evaluation of railway track behavior [3,17]. Although, a number of ϕ-DAS systems capable of measuring relatively large strain levels have recently been demonstrated, the sensing range in none of those studies were exceeding [18,19,20,21].

To address this limitation, several Brillouin-based DAS (B-DAS) systems have been developed capable of measuring large dynamic strains [22,23,24,25,26]. Since the strain measurement in B-DAS systems is based on the changes of the Brillouin Frequency Shift (BFS), B-DAS systems have no limitation in detecting large strain variations so far as they can acquire the Brillouin Gain Spectrum (BGS) at each point on the sensing fiber fast enough. The commonly used interrogation techniques used in B-DAS systems include Brillouin Optical Time-domain Analysis (BOTDA), Brillouin Optical Correlation-domain Analysis (BOCDA), and Brillouin Optical Time-domain Refractometry (BOTDR).

BOTDA interrogation technique is based on the interaction of two counter propagating optical signals, i.e., a pulsed pump signal and Continuous Wave (CW) probe signal, separated in frequency by the BFS of the fiber. Conventional BOTDA interrogators form the BGS by stepping the frequency of the CW probe over a certain frequency range, a time-consuming process which limits the sampling rate of the sensor [27,28]. To overcome this limitation, a technique based on Slope-Assisted (SA) mechanism is introduced that improves the detection bandwidth of BOTDA systems. In this method, the frequency of the probe signal is dynamically changed to match the frequency of the full-width half-maximum (FWHM) point on the BGS curve at each section of the sensing fiber. By locking the frequency of the probe to the quadrature point of the BGS curve, any Brillouin frequency shifts is converted to intensity variations which can be directly measured by detectors [26,29,30,31]. Using this method, vibrations at 400 Hz with spatial resolution of 1.5 m and accuracy of 5 με was measured over a length of 85 m [29]. Several variations of the SA-BOTDA method, such as double/multi-SA BOTDA and BOTDA based on phase-gain ratio, have also been proposed, demonstrating an improved dynamic range, speed and accuracy of this sensing technique. In general, works have been reported demonstrating dynamic ranges of ∼5 mε [31] at typical sampling rates of 1 kHz [32] with spatial resolutions as low as 1 m [31] and a maximum sensing range of 2 km [30,32].

BOCDA is another Brillouin-based interrogation technique that has been used to map dynamic strains along sensing fibers. The operation of BOCDA-based systems is based on generating stimulated Brillouin scattering (SBS) at a single point on the sensing fiber by sinusoidally modulating the frequency of two Brillouin-frequency-shifted CW signals and launching them from two opposite ends of the sensing fiber [32,33]. In their most basic format, BOCDA systems are relatively slow as they require to obtain the BGS along the fiber one point at a time. In order to increase the sensing bandwidth of BOCDA systems, various techniques have been devised. The first technique, named single-SA BOCDA, was inspired by the SA-BOTDA sensing principle in which the frequency-difference between two counter propagating signals is tuned dynamically to satisfy the phase matching condition at each and every section of the sensing fiber. Single-SA BOCDA systems have been used to measure vibrations of up to 100 Hz over 130 m of sensing fiber with a spatial resolution of 3.45 cm [33]. Dual-SA BOCDA is a modified version of single-SA BOCDA interrogation technique capable of measuring dynamic strain with frequencies and amplitude of 625 Hz and 700 με, respectively, over 20 m sensing fiber and with 7 cm spatial resolution [34]. Brillouin Optical Correlation-domain Refractometry (BOCDR) is another correlation-domain interrogation technique but unlike BOCDA, it only requires access to a single-end of the sensing fiber [34,35]. Sensing systems based on BOCDR have been used to measure dynamic events with a strain range and frequency of 3 mε and 100 Hz, respectively, and a spatial resolution of 40 cm over a 12 m-long fiber [34]. Despite their excellent spatial resolutions, systems based on correlation-domain interrogation techniques are best suited for applications that require high spatial resolution dynamic measurement over a relatively short sensing range.

BOTDR is the simplest interrogation technique which is based on direct detection of the spontaneous Brillouin Scattering [22,23,24,25,26,36,37]. Unlike other interrogation methods, this technique requires access to a single end of the sensing fiber which has a great advantage when dealing with broken fibers under test. In their most basic format, however, distributed sensors based on BOTDR interrogation technique are relatively slow as they require forming the BGS at each point of the fiber one frequency at a time [38,39]. Several works have been reported in order to improve the performance of this method. For instance, BOTDR sensors based on the short-time Fourier transform algorithm [40] is used for fast detection of 60 Hz vibrations with a strain amplitude of 3 mε and spatial resolution of 4 m employing small-gain stimulated Brillouin scattering in a 1 km fiber range [24]. This method utilizes coherent down-conversion of the backscattered Brillouin signal and electronic filtering in order to reconstruct the BGS by a short-time Fourier transform algorithm. This leads to a trade-off between spatial and frequency resolutions. Dynamic SA-BOTDR is another interrogation technique presented in [41] demonstrating dynamic strain measurements with a strain resolution of 40 με at 7.6 Hz acquisition rate over 100 m of fiber and with a spatial resolution of 1 m. In this method, a heterodyne detection scheme is employed along with an adaptive local oscillator with a reference fiber (to produce a SBS signal to beat with the backscattered signal) to lock onto the maximum slope of the Brillouin gain spectrum. This limits the dynamic range of the sensor to the linear region of the BGS. Frequency-agile BOTDR is yet another technique based on classical BOTDR system in which a frequency-agile modulated reference wave is mixed with the backscattered Brillouin signal [23]. Using this method, 14.77 Hz vibration with a strain range and resolution of 4 mε and ±30 με, respectively, is measured over a fiber length of 172 m and a spatial resolution of 2 m. Although the strain range of frequency-agile BOTDR is improved compared with the SA-BOTDR technique, the frequency-agile method is limited in terms of the measurement time especially when the number of sweeping frequencies or the fiber length increases. The aim of this study is, first, to show the theoretical limit of ϕ-DAS systems in terms of the maximum detectible strain rate as function of the sensing range and, subsequently, to demonstrate a long-range distributed sensor based on BOTDR interrogation technique capable of measuring dynamic events with a maximum strain frequency and amplitude of 120 Hz and 8.7 mε, respectively over a sensing range of 8.5 km. This sensing range combined with 2.5 με strain sensitivity constitute a factor of 200 improvement from the state-of-the-art B-DAS system. This is achieved by adopting an optical filter based on Mach-Zehnder interferometer (MZI) thus eliminating the need for locking the local oscillator to the linear region of the BGS. In addition, the sensing range of the DAS system is further extended to 20 km, a value which is an order of magnitude longer than longest B-DAS range ever reported [32], by employing an in-line pulsed Raman amplification.

The rest of the paper is organized as follows. In Section 2, limitation of the ϕ-DAS systems is described in terms of the maximum detectable strain rate and the sensing range. Section 3 describes the sensing principal of the B-DAS system. Section 4 explains the experimental setup and sensing procedure of the B-DAS system. Response of the sensor as a function of strain amplitude, range, and frequency is characterized in Section 5, followed by the details of the B-DAS system with extended range based on pulsed Raman in-line amplifier. Finally, results of this work are concluded in Section 6.

## 2. Strain Rate Limitations of ϕ-DAS Systems

In this section, the theoretical limit of ϕ-DAS systems will be analysed to show the trade-off between the sensing range and strain rate of such systems showing the drawback of using ϕ-DAS for measuring events with high strain rate. Operation of ϕ-DAS is based on mixing the Rayleigh backscattered light from adjacent points on the fiber and analyzing the changes in the interference pattern in order to measure the variation in the distance between those points [42]. Each section of the sensing fiber is sampled at a fixed time interval which is determined by the round-trip time of light in the fiber. To avoid signal aliasing, no section of fiber should experience strain-induced phase shift of more than π radians within that time interval. Such constraint translates to a maximum length change of less than λ/2n during the round-trip time of light where λ and *n* denote the wavelength of the probe pulse and effective refractive index of the fiber, respectively. For such DAS systems relying on optical interferometry, the maximum detectible vibration at each point along the fiber depends on both the repetition rate of the probe pulse, $T_{R}$, and the strain level, ε [22]. This relation can be demonstrated by analyzing the equation that governs the behavior of optical interferometer of the DAS system:

(1) $$I=A+B\sin\left(\Delta \varphi \left(t\right)\right)$$

where *I* is the intensity at the output of the MZI, *A* and *B* are constant values, and Δφ(t) is the strain-induced phase change between two points on the sensing fiber at time *t*. For any given section of a fiber, the value of Δφ(t) is given by [43]

(2) $$\Delta \varphi \left(t\right)=0.78\times \frac{4\pi n}{\lambda }\Delta \ell$$

where Δℓ denotes the elongation between two points on the fiber separated by distance *L*. For a sinusoidal strain wave with frequency *f*, the value of Δℓ can be written as

(3) $$\Delta \ell =\varepsilon L\sin\left(2\pi ft\right)$$

where ε is the strain amplitude. Combining Equations (1)–(3) yields

(4) $$I=A+B\sin\left[0.78\times \frac{4\pi n}{\lambda }\varepsilon L\sin\left(2\pi ft\right)\right]$$

**Figure 1:**
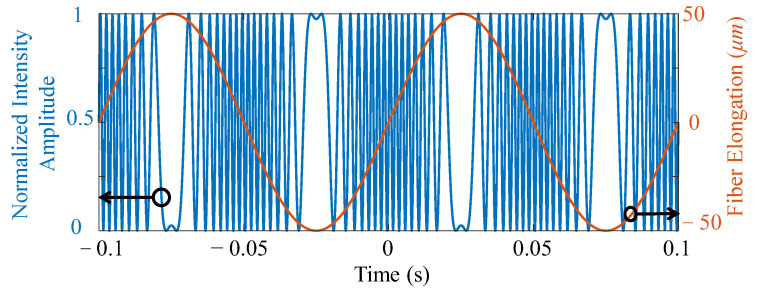
A comparison of the fluctuation frequency of the strain wave on the vibrating fiber Equation (3) and the optical intensity seen at the detector (4) for *f* = 10 Hz and ε·L = 5 $\times \;10^{-5}$ m.

This equation shows that a sinusoidal vibration with frequency *f* may not necessarily translate to an intensity modulation with the same frequency at the detector. As an example, Figure 1 demonstrates the oscillation of the output optical intensity (blue trace) for 10 Hz oscillation with Δℓ = 50 μm (orange trace). This shows that the frequency of the signal at the output of the detector can be several times higher than the strain frequency. Therefore, setting the pulse repetition frequency of a ϕ-DAS system to twice the frequency of the strain wave does not necessarily guarantee signal integrity.

In order to determine the maximum intensity excursion rate at the interferometer output, the derivative of Equation (4) needs to be calculated:

(5) $$\frac{dI}{dt}=B\cos\left[0.78\times \frac{4\pi n}{\lambda }\varepsilon L\sin\left(2\pi ft\right)\right]\times 0.78\times \frac{8\pi^{2}nf}{\lambda }\varepsilon L\cos\left(2\pi ft\right)$$

which exhibits maxima at t=N/f where *N* is an integer. This equation shows that the detector experiences a maximum excursion rate at *t* = 0. Using Maclaurin expansion for sin(2πft) in Equation (4), one can rewrite it such that

(6) $${\left.I\right|}_{t\to 0}≅A+B\sin\left[0.78\times \frac{4\pi n}{\lambda }\varepsilon L\times 2\pi ft\right].$$

According to this Equation, the intensity modulation frequency of the detector for a sinusoidal strain wave with frequency and amplitude of *f* and ε, respectively, peaks at

(7) $$f_{\max}=0.78\times \frac{4\pi n}{\lambda }\varepsilon Lf.$$

Therefore, to avoid temporal aliasing, DAS interrogator should have a minimum pulse repetition rate of

(8) $$T_{R}=\frac{1}{2f_{\max}}=\frac{\lambda }{0.78\times 8\pi n\times \varepsilon Lf}.$$

Analysis of Equation (8) demonstrates a trade-off between the strain rate experienced by the fiber and the repetition rate of the probe pulse. For an OTDR system, the maximum pulse repetition rate is determined by the round-trip time of light in the sensing fiber. For a fiber with length *D*, the maximum repetition rate is

(9) $${\left.T_{R}\right|}_{\max}=\frac{2Dn}{c}$$

where *c* is the speed of light in vacuum. By combining Equations (8) and (9), the relationship between the length of the sensing fiber and the maximum detectible strain rate would be

(10) $$D=\frac{\lambda c}{0.78\times 16\pi n^{2}\times \varepsilon Lf}$$

**Figure 2:**
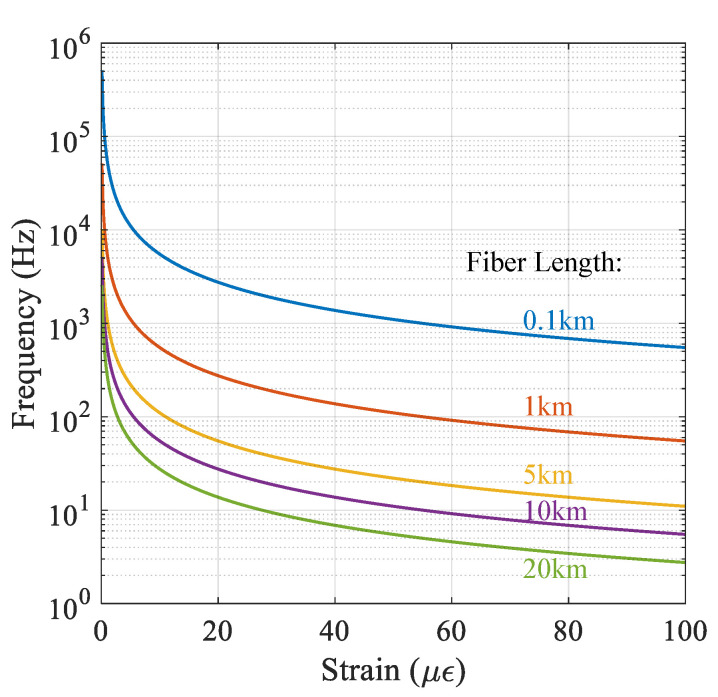
Maximum measurable strain frequency as a function of the strain amplitude for fiber lengths of 0.1, 1, 10, 20 km. The spatial resolution is assumed to be *L* = 1 m.

For a fiber with a given length, this equation puts an upper limit on the strain rate that a ϕ-DAS system can measure without the risk of temporal aliasing. Using Equation (10), the maximum strain frequency that a ϕ-DAS with 1 m spatial resolution (*L* = 1 m) can detect as a function of the strain amplitude is plotted in Figure 2, showing that maximum detectible strain rate is inversely proportional to the length of the fiber. This characteristic can be associated to interferometric response where, for vibrations with high strain rate, the output of the interferometer travels over multiple fringes. Hence, to accurately measure a vibration with a high strain level, a ϕ-DAS should have a sampling rate high enough to count the interferometric fringes and avoid signal aliasing.

## 3. Principal of B-DAS

**Figure 3:**
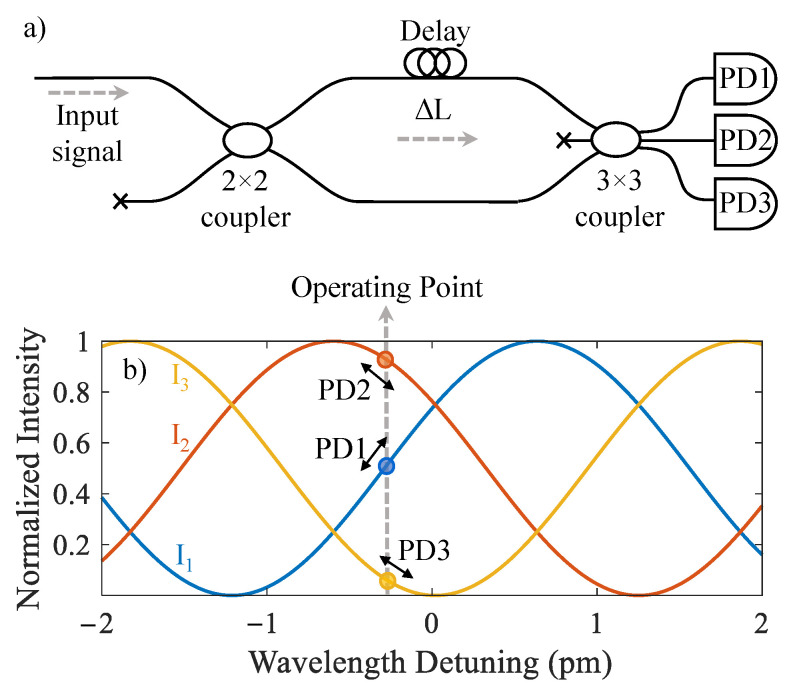
(**a**) Schematic of the MZI filter with a 3 × 3 coupler as the output coupler, and (**b**) transfer function of MZI in terms of the output intensity of the PDs for ΔL = 65 cm and center wavelength of λ = 1550 nm, and example of the sensor operating point.

The frequency transfer function of the imbalanced MZI at the output of the B-DAS system determines the response of the sensor to BFS. A schematic of the MZI used for demodulation of the BFS is shown in Figure 3a. ΔL is the path imbalance and determines the Free Spectral Range (FSR) of the filter. For an input Brillouin signal with wavelength of λ, the output intensity of the three arms of the filter can be written as [22]

(11) $$\begin{array}{cc} \; & I_{1}=\frac{A}{3}\left[1+\cos\left(\frac{2\pi }{\lambda }\Delta L+\frac{2\pi }{3}\right)\right],\\ \; & I_{2}=\frac{A}{3}\left[1+\cos\left(\frac{2\pi }{\lambda }\Delta L\right)\right],\\ \; & I_{3}=\frac{A}{3}\left[1+\cos\left(\frac{2\pi }{\lambda }\Delta L-\frac{2\pi }{3}\right)\right] \end{array}$$

where *A* is the intensity of the light at the input of the MZI. Any changes in the wavelength of the input signal translates into optical intensity variation at the photodetectors. Thus, the strain-induced BFS results in an intensity variation of the light at the three output arms of the MZI. This variation is illustrated in Figure 3b for ΔL = 65 cm and λ = 1550 nm. The output of the three photodetectors can be combined using differentiate and cross-multiply demodulation scheme to extract the dynamic strain information as a function of ΔL and λ [44]

(12) $$\Phi =f\left(I_{1},I_{2},I_{3}\right)=\frac{2\pi }{\lambda }\Delta L=\frac{2\pi \cdot \upsilon }{c}\Delta L$$

where υ is the frequency of the Brillouin backscattered light. This shows that the demodulator transforms the cosine transfer function of the MZI to a quantity proportional to the BFS. Using this arrangement, the maximum step change in the strain level allowed between two samples would be determined by the strain-induced BFS and the FSR of the MZI filter. With BFS strain-coefficient of 20 με/MHz, an MZI filter with FSR of 320 MHz (ΔL = 65 cm) would allow for a maximum strain change of 3.2 mε between each sample. The differentiate and cross-multiply demodulation scheme allows for measurement of strain changes beyond 3.2 mε through fringe counting provision. The strain sensitivity of the sensor is proportional to ΔL if the FSR of the filter is much larger than the bandwidth of the Brillouin signal.

## 4. B-DAS Experimental Setup and Sensing Procedure

### 4.1. Experimental Setup

**Figure 4:**
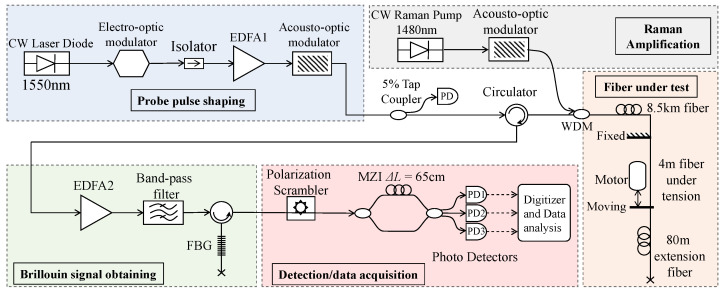
Experimental setup consisting of five sections: probe pulse shaping, fiber under test, scattered Brillouin signal collection, detection/data acquisition and Raman amplification (EDFA: Erbium-doped fiber amplifier, FBG: Fiber Bragg Grating, MZI: Mach Zehnder Interferometer, PD: Photodetector, EOM: Electro-optic modulator, and WDM: Wavelength Division Multiplexer).

Figure 4 depicts the B-DAS setup used in the experiments, which consists of five sections. A 1550 nm semiconductor laser diode with 60 kHz linewidth and 10 mW power was intensity modulated by an electro-optic modulator (EOM) to generate 40 ns pulses at a repetition rate of 1.2 kHz. An Erbium-doped Fiber Amplifier (EDFA1) was used to increase the peak power of the pulse to 370 mW. The amplified probe pulses were then picked by an acousto-optic modulator (AOM) in order to eliminate the residual Amplified Spontaneous Emission (ASE) from EDFA1. A 5% tap coupler was used to monitor the probe pulse. An optical circulator was used to launch the probe pulse into ∼8.5 km of single-mode fiber (Corning SMF-28) of which a 4 m section at the far end was stretched using a custom-made reciprocating actuator developed in-house. An 80 m-long SMF was spliced to the fiber on the actuator to separate the strain-induced section of the fiber under test from the far-end of the sensing fiber.

The backscattered light was collected via an optical circulator and amplified by an optical pre-amplifier (EDFA2). A band-pass Dense Wavelength Division Multiplexing (DWDM) filter with 100 GHz bandwidth and a Fiber Bragg Grating (FBG) with 59% reflectivity and 0.03 nm bandwidth were employed to suppress the ASE and Rayleigh backscattered lights, respectively. The wavelength of the seed laser was tuned to align the frequency of the backscattered Brillouin anti-stokes signal with the central frequency of the FBG filter. The filtered light was then passed through a polarization scrambler to reduce the effect of the polarization dependent components in the MZI on the Brillouin signal. The path imbalance of the MZI was set to ΔL = 65 cm to create a filter with ∼320 MHz FSR [45]. The outputs of the MZI were detected by three Photodetectors (PDs) with 125 MHz bandwidth. A digitizer with 500 MHz bandwidth was used to acquire the output voltages of the PDs. To extend the range of the B-DAS, Raman inline amplification was included in the setup to amplify the probe signal through Raman gain as it propagated down the fiber. A 1480 nm CW Raman fiber laser was pulsed using an AOM and the pulsed light was launched into the test fiber by a 1480/1550 nm WDM. To demonstrate an extended range B-DAS system, the length of the SMF under test was increased to ∼20 km and the peak power of the probe pulse was lowered to ∼250 mW to avoid modulation instability. A co-propagating pulsed Raman pump with 570 mW peak power was used for Raman amplification.

### 4.2. Sensing Procedure

For the tests with 8.5 km fiber, the repetition rate of the probe pulse was set to 1.2 kHz. The Brillouin backscattered light from the fiber under test was filtered by the MZI after pre-amplification. The MZI filter was used to convert the changes in BFS into intensity variations. The short path imbalance of the MZI relative to the duration of the probe pulse guaranteed mixing the backscattered light from the same section of the fiber. For low frequency vibrations (i.e., 10 Hz), to reduce the noise level, the BOTDR traces obtained at the PDs were averaged 100 times to reduce the noise level and then analyzed using differentiate and cross-multiply demodulation scheme to extract the dynamic strain information. This method provides a large dynamic range detection through fringe counting algorithm [44]. A reciprocating motor was used to impose 120 Hz dynamic strain with amplitudes of up to 10 mε on the fiber. For higher frequency vibrations, the averaging times was reduced to 10 to avoid temporal aliasing. Sampling rate of the system is dependent to the measurement length and number of averaging which is limited to $c/2N_{ave}Dn$. For $N_{ave}$ = 10 and sensing range of 8.5 km, the maximum measurable frequency was ∼1.2 kHz which is well above 120 Hz in our experiment. In the Raman inline amplification experiment, a pump pulse-width scanning was conducted to determine the optimum pump width for a given pulse-probe delay to attain a desired amplification gain. In order to characterize the B-DAS system, 11 Hz strain waves of 1 mε amplitude were applied to a fiber section of 4 m at ∼20 km range. Then, the BOTDR traces were obtained by 500 Hz sampling rate at 10 times temporal averaging.

## 5. Experimental Results and Discussion

### 5.1. Characterization of B-DAS

**Figure 5:**
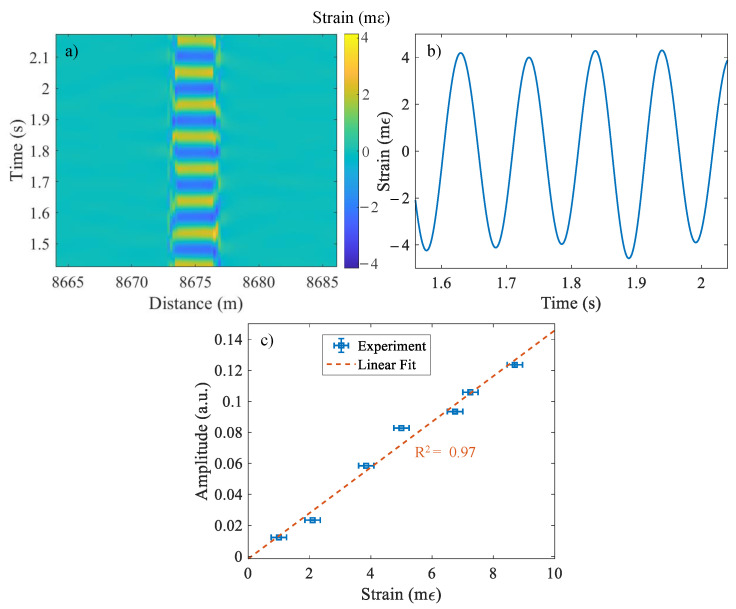
(**a**) Response of the B-DAS system as a function of distance in time for an input sinusoidal strain wave of f ∼10 Hz with a peak-to-peak displacement of 17.2 mm over 4 m of SMF, (**b**) 2D plot of the same response at the middle of the 4 m-fiber, and (**c**) amplitude of the sensor output and its linear fit in terms of the applied peak-to-peak strain at f∼ 10 Hz.

Figure 5a shows the output of the sensor at the far-end of the sensing fiber as a function of time and distance. A sinusoidal strain wave with frequency of ∼10 Hz and an amplitude of 4.15 mε, imposed over 4 m of the SMF, can be observed in this waterfall plot. The 2D cross section of the waterfall plot, shown in Figure 5b, exhibits a periodic oscillation similar to sinusoidal strain exerted on the fiber. Furthermore, Figure 5c shows the amplitude of the sensor output for dynamic strains at a fixed 10 Hz frequency but at various strain levels. A linear fit to the experimental results shows an $R^{2}$ = 0.97. The slight discrepancy between the expected and measured strains stems from the backlashes in the bearings and the long-length of the reciprocating motor-shaft system as well as the nonlinear behavior of the motor under a variable load.

**Figure 6:**
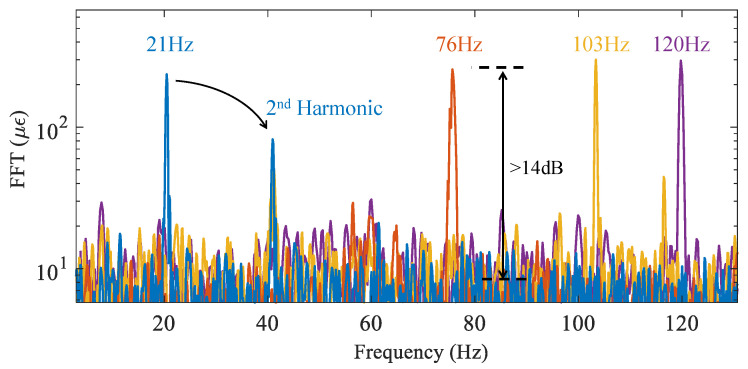
Frequency spectra of the sensor excited with a strain wave of ∼300 με amplitude at *f* = 21, 76, 103 and 120 Hz (FFT: Fast Fourier Transform). Signals are averaged 10 times.

The frequency response of the sensor to higher frequency vibrations at *f* = 21, 76, 103 and 120 Hz are plotted in Figure 6. For this test, the amplitude of the vibration was kept at 300 με. The observed peaks of the Fast Fourier Transform (FFT) at the applied frequencies exhibit similar levels demonstrating a flat frequency response of the B-DAS. An SNR of ∼14 dB was measured which translate to a strain precision of 12 με.

### 5.2. Characterization of Raman-Amplified B-DAS System

**Figure 7:**
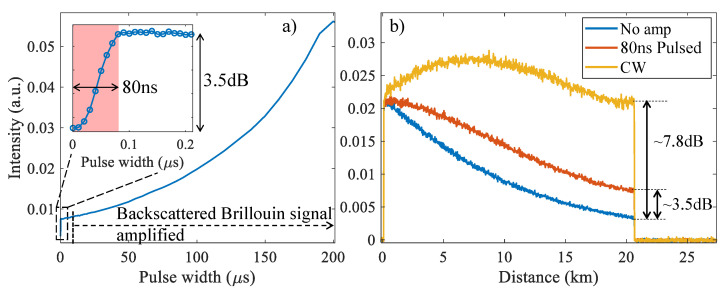
Optical intensity of the BOTDR signal collected before the MZI (**a**) at the end of the 20 km fiber as a function of the Raman pump pulse pulse-width (inset shows detailed view of the plot for very small pulse widths), and (**b**) when there is no Raman amplification, amplified by 80 ns-wide pulse and CW pump.

Figure 7a shows the intensity of the Brillouin backscattered light at the far-end of a 20 km-long fiber as a function of the Raman pump pulse width. To avoid modulation instability, the delay between the copropagating pump and probe pulses was adjusted such that the two pulses reach each other at a desired point along the fiber where the probe pulse was sufficiently attenuated. The experimental results show that, the maximum amplification of ∼3.5 dB was reached at 80 ns pump pulse (see Figure 7a inset).

Due to the different group velocities of signal and pump modes (in anomalous dispersion regime), the amplification was limited to the fiber location where pump and signal pulses spatially overlap. The amplification length can be approximately given by $L_{amp}=cT_{p}/\left(n_{p}-n_{s}\right)$, where $T_{p}$ is the pump pulse-width, $n_{p}$ and $n_{s}$ denote the pump and signal effective refractive indices, respectively. In this relation, the partial overlap of the pulses at the edges are ideally ignored (i.e., the signal is assumed to experience a uniform gain over its width). Accordingly, to achieve a maximum 20 km of interaction length (which corresponds to the fiber length) for a typical value of $n_{p}-n_{s}=10^{-3}$ [46], a pump pulse with a minimum duration of ∼67 ns is required. Since the used pulses were not rectangular and partially overlapped at the edges, the optimum amplification was reached for 80 ns pulse duration. Further increasing the pump pulse width did not amplify the probe pulse due to limited fiber length, and only amplified the backscattered Brillouin signal. At pulse-widths above 4 μs, the amplification of the Raman pump on the backscattered Brillouin signal became significant. Figure 7b compares the obtained BOTDR trace for no amplification, pulsed Raman amplification, and CW Raman amplification. DC levels of the traces are removed to facilitate the comparison between the three scenarios. At the far-end of the fiber, 3.5 dB and 7.8 dB amplifications are observed for BOTDR traces corresponding to pulsed and CW pumps, respectively.

**Figure 8:**
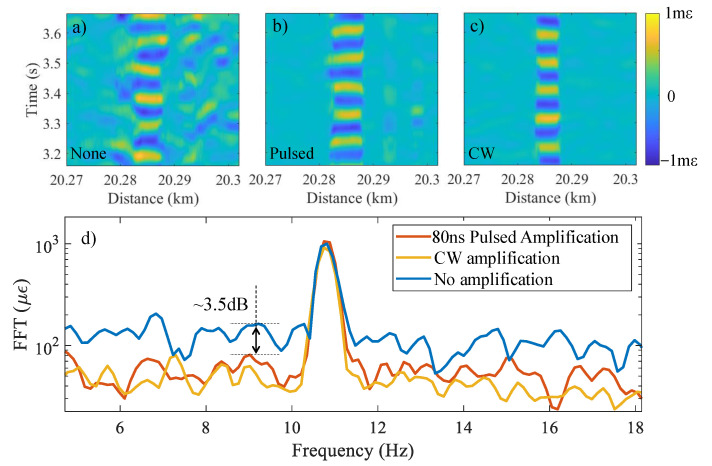
Performance of the DAS as function of distance and time when a sinusoidal strain with 1 mε of amplitude at ∼11 Hz is applied under three conditions: (**a**) no amplification, (**b**) 80 ns-linewidth pulsed, and (**c**) CW Raman amplification. (**d**) Frequency content of the obtained signals in cases (**a**–**c**) demonstrating the noise level.

A comparison of the output of the B-DAS system, operating under various Raman amplification conditions, is shown in Figure 8a–d. A sinusoidal strain wave at an acoustic frequency and amplitude of ∼11 Hz and ∼1 mε, respectively, was applied on the fiber. Figure 8a plots the output of the sensor when no Raman amplification was used. A higher noise level is visible at the far-end of the fiber where strain is applied, resulting in distortion in the detected signal. In contrast, the BOTDR signal amplification by pulsed Raman pump Figure 8b shows a lower noise level and, hence, a better signal integrity. Lastly, CW Raman amplification shows similar SNR as seen in Figure 8c where a clear sinusoidal signal was achieved.

In order to compare the noise level of the three amplification configurations, the FFT of the measured signals are plotted in Figure 8d. A peak at ∼11 Hz is observed for all three amplification scenarios corresponding to the frequency of the applied strain on the fiber. In the case of both pulsed and CW Raman amplifications, the noise floor is 3.5 dB lower compared with the noise floor of the results with no amplification. This corresponds to an improvement in the strain measurement accuracy from 155 με to 62 με. This is in good agreement with the results shown in [47]. Although CW Raman amplification yields BOTDR traces with 4.3 dB higher signal level compared to that of the pulsed Raman amplification (see Figure 7b), the analysis of the noise floors, shown in Figure 8d, indicates that the CW and pulsed Raman amplification yield similar SNR. This is due to the fact that when the Raman pump is off, the main source of the noise is the thermal and shot noises. Whereas, the ASE of the Raman amplifier becomes the dominant noise source when CW Raman pump is used for signal amplification along a long-range fiber [48]. Hence, for 20 km fiber length, Raman amplification with CW pump does not significantly improve the SNR. The drawback of using a CW pump, however, is that it generates backward amplified spontaneous Raman and Rayleigh emissions which requires additional filtering to avoid saturation of the detectors. In addition, launching high-power CW laser into an optical fiber carries a safety risk if the fiber is used for vibration sensing in an environment with explosive materials such as gas pipelines.

## 6. Conclusions

A long-range B-DAS system capable of measuring vibrations with high strain rate was demonstrated. The proposed method provides a sensing system based on interferometric demodulation which does not suffer from intensity fading and does not require any locking mechanism associated with other types of B-DASs [29,30,31]. The behavior of conventional ϕ-DAS systems was theoretically analyzed showing that the frequency of the intensity modulation at the output of these systems can be much higher than the frequency of the dynamic strain experienced by the sensing fiber. As a result, for a given fiber length, the maximum measurable strain rate is limited due to temporal aliasing. To address the limitation of ϕ-DAS systems, a distributed system based on the frequency shift of Brillouin backscattered light was proposed. Using a MZI filter as a frequency-to-intensity convertor, a B-DAS can achieve a dynamic range and frequency of 8.7 mε and 120 Hz, respectively, at the far-end of 8.5 km. The strain resolution of the system was measured to be 12 με over 4 m spatial resolution. The maximum measured strain was only limited by the yield strength of the sensing fiber. Furthermore, the sensing span of the B-DAS system was extended to 20 km through Raman inline amplification. By adopting the inline amplifier, 3.5 dB amplification on the BOTDR trace intensity is observed for 80 ns pump pulse with 570 mW peak power. Using this scheme, the SNR associated with a measured strain wave with a frequency of 11 Hz showed 3.5 dB improvement and a strain accuracy of ∼62 με.

## Data Availability

Data contained in this paper is openly available from the University of Southampton repository at DOI/10.5258/SOTON/D2065.

## References

[1] Ding Z.W., Zhang X.P., Zou N.M., Xiong F., Song J.Y., Fang X., Wang F., Zhang Y.X. (2021). Phi-OTDR Based On-Line Monitoring of Overhead Power Transmission Line. J. Light. Technol..

[2] Masoudi A., Pilgrim J.A., Newson T.P., Brambilla G. (2019). Subsea Cable Condition Monitoring With Distributed Optical Fiber Vibration Sensor. J. Light. Technol..

[3] Milne D., Masoudi A., Ferro E., Watson G., Le Pen L. (2020). An analysis of railway track behaviour based on distributed optical fibre acoustic sensing. Mech. Syst. Signal. Process..

[4] Xu S., Qin Z., Zhang W., Xiong X. (2020). Monitoring Vehicles on Highway by Dual-Channel phi-OTDR. Appl. Sci..

[5] Lellouch A., Yuan S., Spica Z., Biondi B., Ellsworth W.L. (2019). Seismic Velocity Estimation Using Passive Downhole Distributed Acoustic Sensing Records: Examples From the San Andreas Fault Observatory at Depth. J. Geophys. Res. Solid Earth.

[6] Zhu T., Shen J., Martin E.R. (2021). Sensing Earth and environment dynamics by telecommunication fiber-optic sensors: An urban experiment in Pennsylvania, USA. Solid Earth.

[7] Hicke K., Eisermann R., Chruscicki S. (2019). Enhanced Distributed Fiber Optic Vibration Sensing and Simultaneous Temperature Gradient Sensing Using Traditional C-OTDR and Structured Fiber with Scattering Dots. Sensors.

[8] Redding B., Murray M.J., Davis A., Kirkendall C. (2019). Quantitative amplitude measuring phi-OTDR using multiple uncorrelated Rayleigh backscattering realizations. Opt. Express.

[9] Hu Y., Meng Z., Zabihi M., Shan Y., Fu S., Wang F., Zhang X., Zhang Y., Zeng B. (2019). Performance Enhancement Methods for the Distributed Acoustic Sensors Based on Frequency Division Multiplexing. Electronics.

[10] Chen W., Jiang J., Wang S., Liu K., Ma Z., Liang G., Ding Z., Zhang Y., Niu P., Liu T. (2020). Hybrid demodulation method for distributed acoustic sensing based on coherent detection and pulse pair. Appl. Phys. Express.

[11] Ju Z., Yu Z., Hou Q., Lou K., Chen M., Lu Y., Meng Z. (2020). Low-noise and high-sensitivity Phi-OTDR based on an optimized dual-pulse heterodyne detection scheme. Appl. Opt..

[12] Wakisaka Y., Iida D., Oshida H., Honda N. (2021). Fading Suppression of phi-OTDR With the New Signal Processing Methodology of Complex Vectors Across Time and Frequency Domains. J. Light. Technol..

[13] Redding B., Murray M.J., Donko A., Beresna M., Masoudi A., Brambilla G. (2020). Low-noise distributed acoustic sensing using enhanced backscattering fiber with ultra-low-loss point reflectors. Opt. Express.

[14] Sagues M., Pineiro E., Cerri E., Minardo A., Eyal A., Loayssa A. (2021). Two-wavelength phase-sensitive OTDR sensor using perfect periodic correlation codes for measurement range enhancement, noise reduction and fading compensation. Opt. Express.

[15] Rao Y., Wang Z., Wu H., Ran Z., Han B. (2021). Recent Advances in Phase-Sensitive Optical Time Domain Reflectometry (phi-OTDR). Photonic Sens..

[16] Wang Z., Lu B., Ye Q., Cai H. (2020). Recent Progress in Distributed Fiber Acoustic Sensing with Phi-OTDR. Sensors.

[17] Lellouch A., Biondi B.L. (2021). Seismic Applications of Downhole DAS. Sensors.

[18] Bhatta H.D., Costa L., Garcia-Ruiz A., Fernandez-Ruiz M.R., Martins H.F., Tur M., Gonzalez-Herraez M. (2019). Dynamic Measurements of 1000 Microstrains Using Chirped-Pulse Phase-Sensitive Optical Time-Domain Reflectometry. J. Light. Technol..

[19] Zhang L., Yang Z., Gorbatov N., Davidi R., Galal M., Thevenaz L., Tur M. (2020). Distributed and dynamic strain sensing with high spatial resolution and large measurable strain range. Opt. Lett..

[20] Zabihi M., Chen Y., Zhou T., Liu J., Shan Y., Meng Z., Wang F., Zhang Y., Zhang X., Chen M. (2019). Continuous Fading Suppression Method for phi-OTDR Systems Using Optimum Tracking Over Multiple Probe Frequencies. J. Light. Technol..

[21] Coscetta A., Minardo A., Zeni L. (2020). Distributed Dynamic Strain Sensing Based on Brillouin Scattering in Optical Fibers. Sensors.

[22] Masoudi A., Belal M., Newson T.P. (2013). Distributed dynamic large strain optical fiber sensor based on the detection of spontaneous Brillouin scattering. Opt. Lett..

[23] Wang B., Hua Z., Pang C., Zhou D., Ba D., Lin D., Dong Y. (2020). Fast Brillouin Optical Time-Domain Reflectometry Based on the Frequency-Agile Technique. J. Light. Technol..

[24] Shangguan M., Wang C., Xia H., Shentu G., Dou X., Zhang Q., Pan J.W. (2017). Brillouin optical time domain reflectometry for fast detection of dynamic strain incorporating double-edge technique. Opt. Commun..

[25] Li B., Luo L., Yu Y., Soga K., Yan J. (2017). Dynamic Strain Measurement Using Small Gain Stimulated Brillouin Scattering in STFT-BOTDR. IEEE Sens. J..

[26] Ba D., Wang B., Li T., Li Y., Zhou D., Dong Y. (2020). Fast Brillouin optical time-domain reflectometry using the optical chirp chain reference wave. Opt. Lett..

[27] Soto M.A., Thevenaz L. (2013). Modeling and evaluating the performance of Brillouin distributed optical fiber sensors. Opt. Express.

[28] Thevenaz L., Mafang S.F., Lin J. (2013). Effect of pulse depletion in a Brillouin optical time-domain analysis system. Opt. Express.

[29] Peled Y., Motil A., Yaron L., Tur M. (2011). Slope-assisted fast distributed sensing in optical fibers with arbitrary Brillouin profile. Opt. Express.

[30] Zheng H., Feng D., Zhang J., Zhu T., Bai Y., Qu D., Huang X., Qiu F. (2019). Distributed vibration measurement based on a coherent multi-slope-assisted BOTDA with a large dynamic range. Opt. Lett..

[31] Zhou D., Dong Y., Wang B., Jiang T., Ba D., Xu P., Zhang H., Lu Z., Li H. (2017). Slope-assisted BOTDA based on vector SBS and frequency-agile technique for wide-strain-range dynamic measurements. Opt. Express.

[32] Yang G., Fan X., He Z. (2017). Strain Dynamic Range Enlargement of Slope-Assisted BOTDA by Using Brillouin Phase-Gain Ratio. J. Light. Technol..

[33] Wang Y., Zhao L., Zhang M., Zhang J., Qiao L., Wang T., Gao S., Zhang Q., Wang Y. (2020). Dynamic strain measurement by a single-slope-assisted chaotic Brillouin optical correlation-domain analysis. Opt. Lett..

[34] Mizuno Y., Hayashi N., Fukuda H., Nakamura K. (2017). Single-end-access distributed strain sensing with wide dynamic range using higher-speed Brillouin optical correlation-domain reflectometry. Jpn. J. Appl. Phys..

[35] Mizuno Y., Hayashi N., Fukuda H., Song K.Y., Nakamura K. (2016). Ultrahigh-speed distributed Brillouin reflectometry. Light Sci. Appl..

[36] De Souza K., Newson T.P. (2000). Brillouin-based fiber-optic distributed temperature sensor with optical preamplification. Opt. Lett..

[37] Kee H.H., Lees G.P., Newson T.P. (2000). All-fiber system for simultaneous interrogation of distributed strain and temperature sensing by spontaneous Brillouin scattering. Opt. Lett..

[38] Maughan S.M., Kee H.H., Newson T.P. (2001). 57-km single-ended spontaneous Brillouin-based distributed fiber temperature sensor using microwave coherent detection. Opt. Lett..

[39] Alahbabi M.N., Lawrence N.P., Cho Y.T., Newson T.P. (2004). High spatial resolution microwave detection system for Brillouin-based distributed temperature and strain sensors. Meas. Sci. Technol..

[40] Tu G., Zhang X., Zhang Y., Ying Z., Lv L. (2014). Strain variation measurement with short-time Fourier transform-based Brillouin optical time-domain reflectometry sensing system. Electron. Lett..

[41] Maraval D., Gabet R., Jaouen Y., Lamour V. (2017). Dynamic Optical Fiber Sensing With Brillouin Optical Time Domain Reflectometry: Application to Pipeline Vibration Monitoring. J. Light. Technol..

[42] Masoudi A., Newson T.P. (2016). Contributed Review: Distributed optical fibre dynamic strain sensing. Rev. Sci. Instrum..

[43] Chen M., Masoudi A., Brambilla G. (2019). Performance analysis of distributed optical fiber acoustic sensors based on phi-OTDR. Opt. Express.

[44] Masoudi A., Belal M., Newson T.P. (2013). A distributed optical fibre dynamic strain sensor based on phase-OTDR. Meas. Sci. Technol..

[45] De Souza K., Newson T. (2001). Improvement of signal-to-noise capabilities of a distributed temperature sensor using optical preamplification. Meas. Sci. Technol..

[46] Malitson I.H. (1965). Interspecimen Comparison of the Refractive Index of Fused Silica. J. Opt. Soc. Am..

[47] Nuno J., Martins H.F., Martin-Lopez S., Ania-Castanon J.D., Gonzalez-Herraez M. (2021). Distributed Sensors Assisted by Modulated First-Order Raman Amplification. J. Light. Technol..

[48] Bromage J. (2004). Raman Amplification for Fiber Communications Systems. J. Light. Technol..

